# Glycosylation Variability of Serum α1-Acid Glycoprotein in the Context of Developing Inflammation and Oxidative Stress in Patients with Severe COVID-19

**DOI:** 10.3390/ijms262210946

**Published:** 2025-11-12

**Authors:** Ewa Maria Kratz, Patrycja Kossakowska, Izabela Kokot, Violetta Dymicka-Piekarska

**Affiliations:** 1 Division of Laboratory Diagnostics, Department of Laboratory Diagnostics, Wroclaw Medical University, Borowska Street 211A, 50-556 Wroclaw, Poland; patrycja.kossakowska@yahoo.com (P.K.); izabela.kokot@umw.edu.pl (I.K.); 2 Department of Clinical Laboratory Diagnostics, Medical University of Bialystok, Waszyngtona Street 15A, 15-269 Bialystok, Poland; violetta.dymicka-piekarska@umb.edu.pl

**Keywords:** COVID-19 (coronavirus disease 2019), serum AGP (α1-acid glycoprotein) glycosylation, inflammation, lectin-ELISA, oxidation-reduction potential

## Abstract

In COVID-19 (coronavirus disease 2019), multi-organ complications depend on the immune system’s activity. α1-Acid glycoprotein (AGP) is a highly glycosylated positive acute-phase protein having multifaceted immunomodulatory and protective effects. We were interested in changes in serum AGP concentrations, expression of its glycans, and oxidation-reduction potential (ORP) between severe COVID-19 patients, convalescents, and healthy controls, and whether any of the analyzed parameters could serve as an additional diagnostic biomarker of severe COVID-19 and/or help monitor recovery. We were also interested in associations between the examined parameters. AGP concentrations were measured using an immunoturbidimetric method. The profile and degree of AGP glycosylation were analyzed using lectin-ELISA with lectins: sialo-specific from *Sambucus nigra* (SNA) and *Maackia amurensis* (MAA), fucose-specific from *Lotus tetragonolobus* (LTA) and *Aleuria aurantia* (AAL). The static and capacitive ORP (sORP and cORP, respectively) were measured using MiOXSYS C+^®^ device (Caerus Biotechnologies, Vilnius, Lithuania). Statistica13.3PL software was used for statistical analysis. AGP concentrations increased in COVID-19 patients, showing high clinical usefulness in distinguishing them from convalescents and controls. AGP α2,6-sialylation (reactivity with SNA) was reduced in COVID-19 vs. other study groups, while α2,3-sialylation (reactivity with MAA) was reduced in convalescents vs. controls. The expression of LTA-reactive fucose (Lewis^x^ structures, Le^x^) was reduced in COVID-19 patients compared to controls and convalescents, but AGP reactivity with AAL did not differ between the study groups. The sORP was reduced, and the cORP was increased in COVID-19. The observed negative correlations between sORP and AGP levels may suggest the antioxidant effect of AGP during severe COVID-19. Higher levels of serum AGP in severe COVID-19, together with low expression of sialic acid α2,6-linked and Le^x^ structures, accompanied by reduced sORP, constitute a characteristic pattern of biomarker expression during severe COVID-19. The increased expression of SNA-reactive sialic acid and Le^x^ structures may reflect the recovery process after SARS-CoV-2 (severe acute respiratory syndrome coronavirus 2) infection. The observed negative correlations between AGP and sORP levels may suggest that serum AGP in COVID-19 also plays a role as an antioxidative molecule.

## 1. Introduction

Coronavirus disease 2019 (COVID-19), caused by SARS-CoV-2 (severe acute respiratory syndrome coronavirus 2), was first identified in China in December 2019, triggering a global pandemic that has significantly impacted our lives over several years, ranging from social to economic aspects [1]. Approximately 20% of COVID-19 patients develop symptoms of lower respiratory tract involvement and multi-organ complications, the pathophysiology of which is largely dependent on the immune system’s activity. Paradoxically, it is not the virus itself, but the body’s own immune response mechanisms that are the main factor disrupting the proper functioning of the human body [2].

α1-Acid glycoprotein (AGP, also known as orosomucoid, ORM) is a positive acute-phase protein with proven multifaceted immunomodulatory and protective effects. AGP belongs to the lipocalin family, has a molecular mass of 37–54 kDa, a length of 183 amino acids, and a pI in the range of 2.8–3.8. AGP is synthesized primarily in hepatocytes and secreted into blood plasma, where, under physiological conditions, it is present at a concentration of 40–120 mg/dL. In pathological conditions, its concentration increases 1–10-fold, depending on the severity of the disease and the presence of factors that stimulate its secretion [3,4]. AGP level is mainly controlled by a combination of inflammatory mediators such as glucocorticoids, TNF-α (tumor necrosis factor α), and proinflammatory interleukins (ILs) such as IL-1, IL-6, and IL-11 [3,5,6,7]. AGP is one of the most highly N-glycosylated serum proteins—it has five N-glycosylation sites and approximately 45% of the molecule’s mass consists of highly heterogeneous N-glycans, including those containing sialic acid (SA) residues, which affects the properties of this protein, such as its low pI value and high solubility in water. The qualitative and quantitative composition of AGP glycans influences the function of this protein and changes depending on the pathophysiological state of the body. It has been demonstrated that specific alterations in the composition of AGP N-glycans, including increased or decreased sialylation or fucosylation, as well as changes in the degree of their branching, are characteristic of certain disease entities [3,4].

An integral part of aerobic respiration, crucial for efficient ATP synthesis, is the production of reactive oxygen species (ROS). These compounds, often free radicals, have found physiological applications throughout evolution as components of defense and signaling mechanisms and in maintaining homeostasis. However, their reactivity carries the risk of damaging key cellular components such as DNA, cell membrane lipids, structural proteins, and enzymes [8,9]. Therefore, studies that expand the knowledge about protective mechanisms and antioxidant factors that neutralize the generated ROS are crucial. Under physiological conditions, the production and neutralization of ROS remain in a tightly regulated balance, also known as redox homeostasis. Weakening the body’s protective mechanisms or increasing the production of ROS, for example, by exogenous factors (e.g., some medications), causes a disturbance to this balance, which is called oxidative stress [8,9,10].

Analysis of both serum AGP concentrations and the profile and degree of its N-glycosylation, supplemented by measurement of the redox potential in the sera of patients with severe COVID-19, convalescents, and healthy individuals who were not infected with SARS-CoV-2 before the study, may contribute to a deeper understanding of the molecular mechanisms underlying the development of many inflammatory diseases, including viral diseases such as COVID-19, and provide a basis for proposing new biomarkers with potential diagnostic applications. Analyzing the variability of the profile and degree of glycosylation of glycoproteins present in body fluids is an interesting, yet underappreciated field of science, offering broad opportunities for further research aimed at both identifying diagnostic biomarkers for many inflammatory diseases and providing a more detailed understanding of the molecular mechanisms based on interactions between glycans and their endogenous ligands that underlie many diseases, including infectious diseases. This study aimed to analyze serum AGP concentrations and the profile and degree of its N-glycosylation in patients with severe COVID-19, in convalescents, and healthy controls who had never been previously infected with SARS-CoV-2. Another aim of our work was to determine whether there were significant differences between the study groups in both AGP concentrations and the expression of its N-glycans, as well as whether there were correlations between the examined parameters. An additional aspect of the study involved measuring the redox potential (oxidation-reduction potential, ORP) in the blood sera of study participants, relating the obtained values to AGP concentrations and AGP glycans expression, and determining whether there are significant differences in values of this parameter between the study groups. Serum AGP concentrations were determined using a turbidimetric method on a biochemical analyzer Konelab20i^®^, while AGP N-glycosylation analysis was based on a modified semi-quantitative solid-phase lectin-ELISA method, using biotinylated lectins specific for sialic acid: SNA (*Sambucus nigra* agglutinin) and MAA (*Maackia amurensis* agglutinin), and fucose: LTA (*Lotus tetragonolobus* agglutinin) and AAL (*Aleuria aurantia* lectin). Oxidation-reduction potential was measured using the MiOXSYS C+^®^ device.

## 2. Results

**Figure 1:**
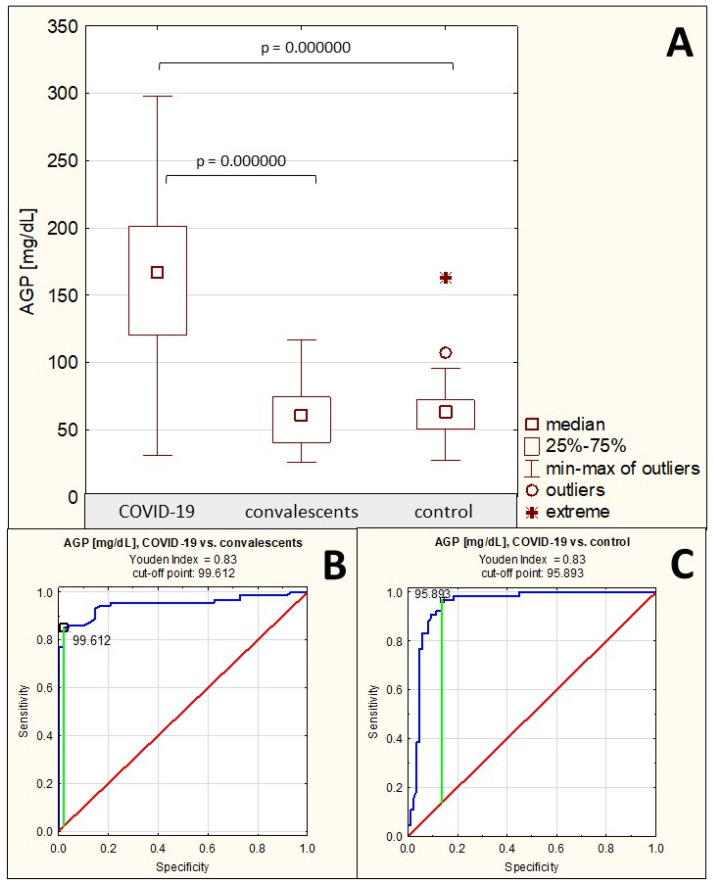
Serum AGP concentrations in individual study groups and results of ROC curve analysis. (**A**)—comparison of serum AGP concentration values between individual study groups; (**B**)—ROC curve for AGP concentration values in the severe COVID-19 patient group and the convalescents; (**C**)—ROC curve for AGP concentration values in the severe COVID-19 patient group and the healthy control group; *p*—significance coefficient (probability value). Values with *p* < 0.05 were considered significant. The green line indicates the optimal cut-off point, the red line indicates the reference line, and the blue line indicates the ROC curve.

**Figure 2:**
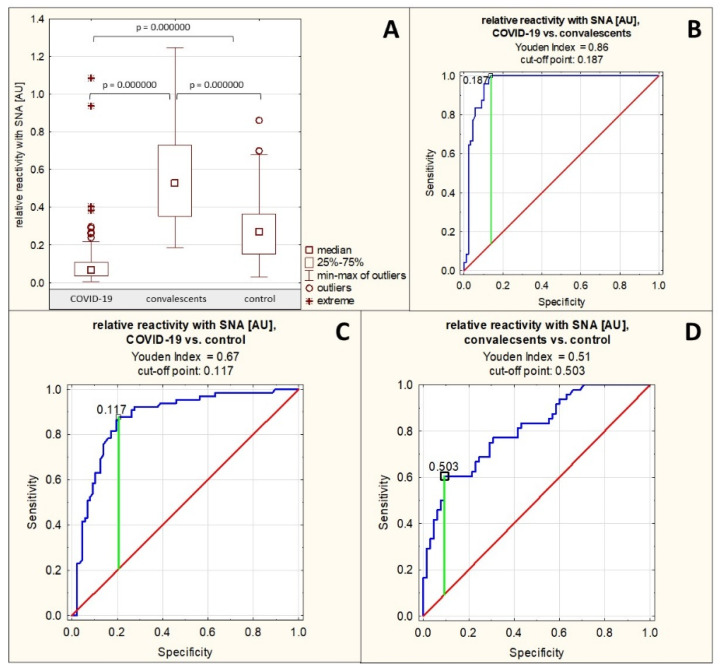
Relative reactivity of serum AGP glycans with SNA in individual study groups and results of ROC curve analysis. (**A**)—comparison of relative reactivity of AGP glycans with SNA in individual study groups; (**B**)—ROC curve for the relative reactivity of AGP glycans with SNA for the group of patients with severe COVID-19 and convalescents; (**C**)—ROC curve for the relative reactivity of AGP glycans with SNA for the group of patients with severe COVID-19 and the healthy control group; (**D**)—ROC curve for the relative reactivity of AGP glycans with SNA for the group of convalescents and the healthy control group; AU—absorbance unit; *p*—significance coefficient (probability value); SNA—*Sambucus nigra* agglutinin. Lectin specificity is given in Section 4. Values for which *p* < 0.05 were considered significant. The green line indicates the optimal cut-off point, the red line indicates the reference line, and the blue line shows the course of the ROC curve.

**Figure 3:**
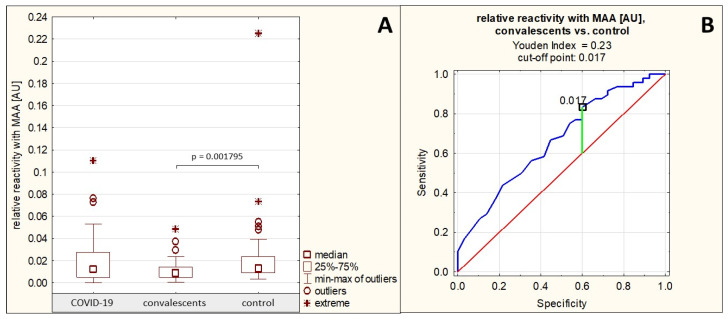
Relative reactivities of serum AGP glycans with MAA in individual study groups and results of ROC curve analysis. (**A**)—comparison of the relative reactivities of AGP glycans with MAA in individual study groups; (**B**)—ROC curve for the relative reactivities of AGP glycans with MAA for the convalescent group and the healthy control group; AU—absorbance unit; *p*—significance coefficient (probability value); MAA—*Maackia amurensis* agglutinin. Lectin specificity is given in Section 4. Values for which *p* < 0.05 were considered significant. The green line indicates the optimal cut-off point, the red line indicates the reference line, and the blue line indicates the ROC curve.

**Figure 4:**
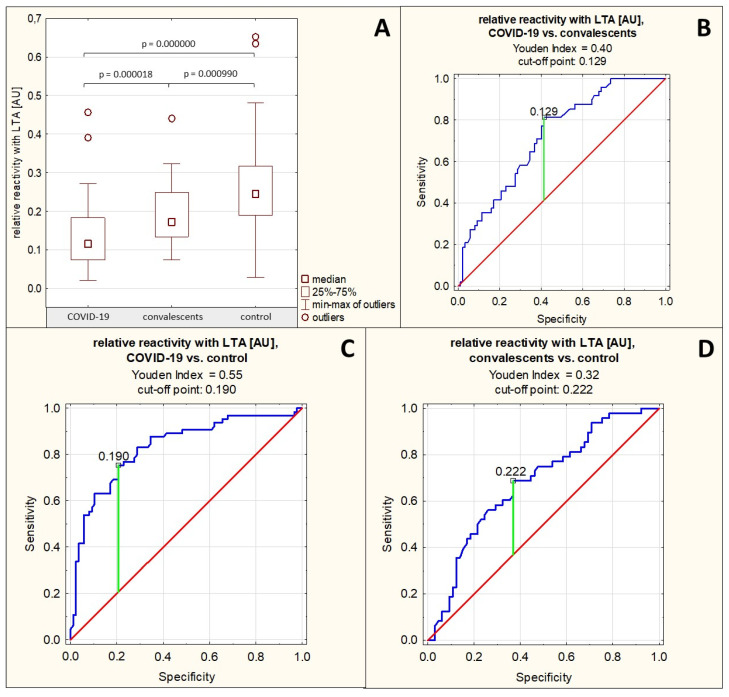
Relative reactivities of AGP glycans with LTA in individual study groups and results of ROC curve analysis. (**A**)—comparison of relative reactivities of AGP glycans with LTA between individual study groups; (**B**)—ROC curve for relative reactivities of AGP glycans with LTA for the group of patients with severe COVID-19 and convalescents; (**C**)—ROC curve for relative reactivities of AGP glycans with LTA for the group of patients with severe COVID-19 and the healthy control group; (**D**)—ROC curve for relative reactivities of AGP glycans with LTA for the group of convalescents and the control group; AU—absorbance unit; *p*—significance coefficient (probability value); LTA—*Lotus tetragonolobus* agglutinin. Lectin specificity is given in Section 4. Values with *p* < 0.05 were considered significant. The green line marks the optimal cut-off point; the red line marks the reference line, and the blue line marks the ROC curve.

**Figure 5:**
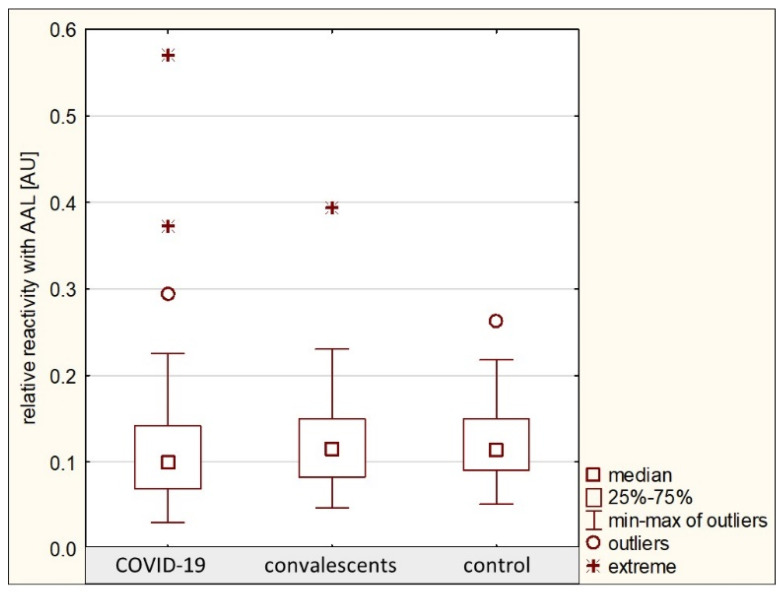
Relative reactivities of AGP glycans with AAL in individual study groups. AAL—*Aleuria aurantia* lectin; AU—absorbance unit. Lectin specificity is given in Section 4.

**Figure 6:**
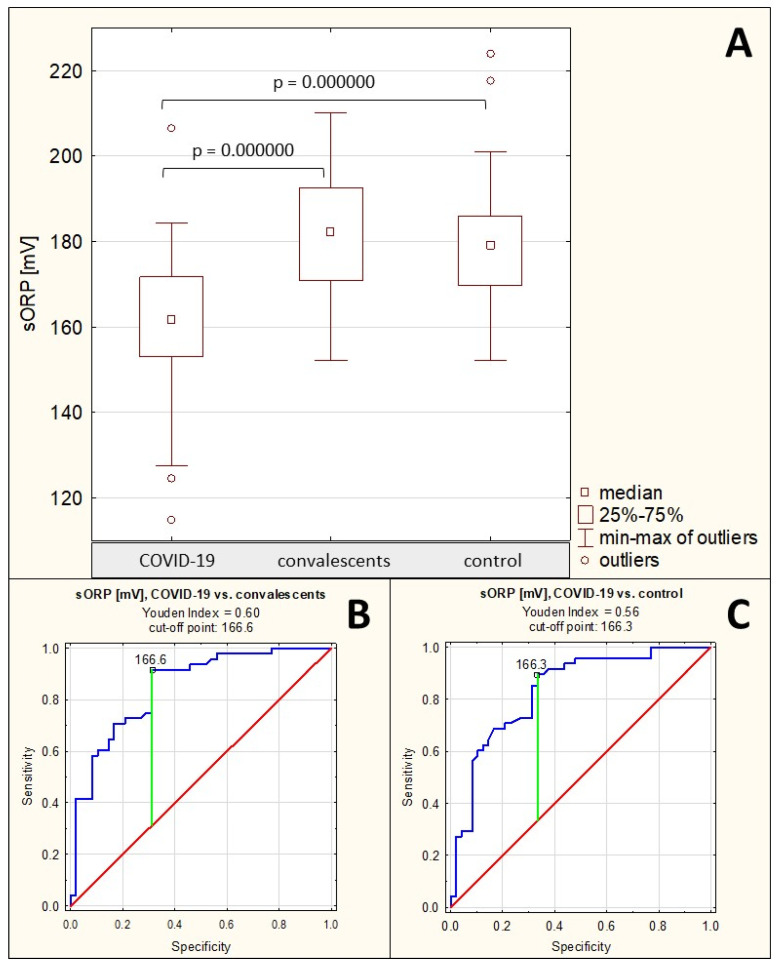
Static redox potential measurement values. (**A**)—comparison of static redox potential (sORP) values between the individual study groups; (**B**)—ROC curve for sORP values for the group of patients with severe COVID-19 and the group of convalescents; (**C**)—ROC curve for sORP values for the group of patients with severe COVID-19 and the group of healthy individuals; *p*—significance coefficient (probability value). Values for which *p* < 0.05 were considered significant. The green line indicates the optimal cut-off point; the red line indicates the reference line, and the blue line indicates the ROC curve.

**Figure 7:**
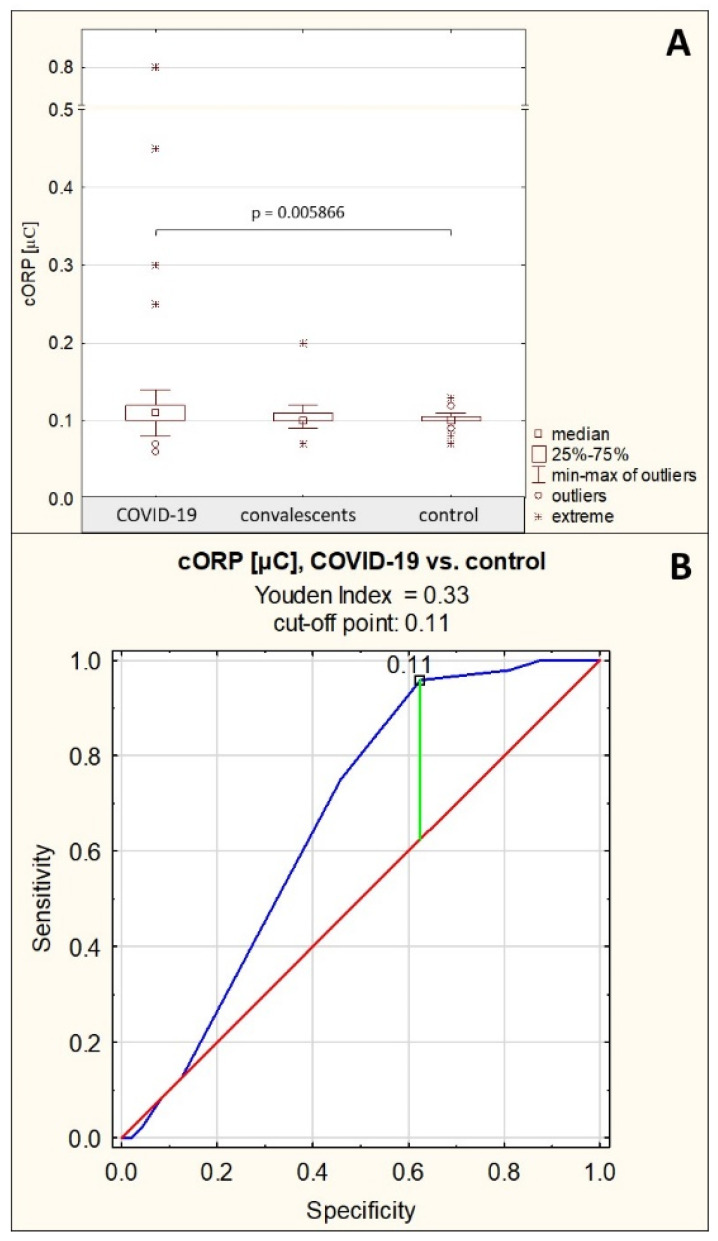
Capacitive redox potential values. (**A**)—comparison of capacitive oxidation-reduction potential (cORP) values between the individual study groups; (**B**)—ROC curve for cORP values for the group of patients with severe COVID-19 and the group of healthy controls; *p*—significance coefficient (probability value). Values for which *p* < 0.05 were considered significant. The green line indicates the optimal cut-off point, the red line indicates the reference line, and the blue line indicates the ROC curve.

In Table 1 are presented the mean values, standard deviations, medians, and *p*-values for all examined parameters in each of the three study groups. These are also compared graphically in Figure 1, Figure 2, Figure 3, Figure 4, Figure 5, Figure 6 and Figure 7, highlighting the median, interquartile range, range of min-max of outliers, and extreme values for each parameter.

Table 2 summarizes the significant (*p* < 0.05) correlations between the analyzed parameter values, specifying the combination of these parameters, Spearman’s rank coefficient (r), *p*-values, and the strength and direction of the correlation.

Table 3 and Table 4 show the results of ROC (receiver operating curve) analyses for the parameter values for which significant (*p* < 0.05) differences were observed between the study participant groups. For these parameters, the AUC (area under the curve) values with 95% confidence interval, the optimal cut-off value determined using the Youden Index, the sensitivity and specificity calculated for the proposed cut-off value, and the probability value ‘*p*’ are provided.

### 2.1. Serum AGP Concentrations

The mean serum AGP concentration in the group of patients with severe COVID-19 was 165.06 ± 58.96 mg/dL (median: 166.81 mg/dL). In the convalescent group and the control group of healthy subjects, the mean AGP concentrations were 61.65 ± 21.77 mg/dL (median: 60.53 mg/dL) and 63.50 ± 21.39 mg/dL (median: 63.08 mg/dL), respectively. Serum AGP concentrations in patients with severe COVID-19 were significantly elevated compared to both the control group (*p* = 0.000000) and the convalescent group (*p* = 0.000000). There were no significant differences in AGP concentrations between the convalescent group and the control group (*p* > 0.05) (Table 1, Figure 1A). Spearman’s rank correlation test revealed: (1) a very high negative correlation between AGP concentrations and the relative reactivities of AGP glycans with the sialo-specific lectin SNA (r = −0.76, *p* = 0.000000), (2) a high negative correlation between AGP concentrations and the relative reactivities of AGP glycans with the fucose-specific lectin LTA (r = −0.53, *p* = 0.000000), (3) a weak negative correlation between AGP concentrations and the relative reactivities of AGP glycans with the fucose-specific lectin AAL (r = −0.28, *p* = 0.000050), (4) a moderate negative correlation between AGP concentrations and static redox potential (r = −0.36, *p* = 0.000009) (Table 2). No significant correlations were found between AGP concentrations and the relative reactivities of its glycans with the sialo-specific lectin MAA (*p* > 0.05).

ROC curves for AGP concentrations are presented in Figure 1B,C. For the severe COVID-19 patients and convalescents, the AUC value was 0.949 (95% confidence interval: 0.913–0.986, *p* = 0.0000, proposed cut-off value was 99.612 mg/dL, with a sensitivity of 85.1% and a specificity of 97.9%), and for the COVID-19 group and the control group, the AUC value was 0.945 (95% confidence interval: 0.905–0.985, *p* = 0.0000, proposed cut-off point was 95.893 mg/dL, with a sensitivity of 96.9% and a specificity of 86.9%) (Table 3), indicating the high clinical value of this parameter.

### 2.2. AGP Sialylation

Sialylation of AGP N-glycans was assessed semi-quantitatively in a lectin-ELISA assay using two lectins: SNA and MAA, which differ in their specificity: SNA detects terminal sialic acid attached to Gal of the sugar antenna via an α2,6 bond, whereas MAA reacts specifically with terminal sialic acid attached to Gal via an α2,3 bond. The relative reactivity of AGP glycans with SNA in the group of patients with severe COVID-19 was, on average, 0.110 ± 0.160 AU (median: 0.066 AU). In the convalescent group, reactivity with this lectin was 0.553 ± 0.259 AU (median: 0.529 AU), and in the control group, 0.289 ± 0.168 AU (median: 0.268 AU). The relative reactivity of AGP glycans with SNA differed significantly between patients with severe COVID-19 and convalescents, as well as between the severe COVID-19 patients and convalescents vs. control group (*p* = 0.000000 for all three comparisons). In the group of patients with severe COVID-19, relative reactivity with this lectin was significantly reduced, whereas in the convalescent group, it was significantly increased compared to the control group (Table 1, Figure 2A). Spearman’s rank correlation test revealed a very high negative correlation between the relative reactivities of AGP glycans with SNA and AGP concentrations as it as mentioned in Section 2.1, a moderate positive correlation between the relative reactivities of AGP glycans with SNA and the relative reactivities with LTA (r = 0.41, *p* = 0.000000), and a weak positive correlation between the relative reactivities of AGP glycans with SNA and the relative reactivities with AAL (r = 0.18, *p* = 0.008836) (Table 2). No significant correlations were found between the relative reactivities of AGP glycans with both sialo-specific lectins (*p* > 0.05).

An analysis of the ROC curves for the group of patients with severe COVID-19 and convalescents (Table 3, Figure 2B) showed that the AUC value for the relative reactivity of AGP glycans with SNA was 0.960 (95% confidence interval: 0.926–0.993, *p* = 0.0000, proposed cut-off point: 0.187 AU, with a sensitivity of 100% and a specificity of 86.2%), which indicates the high clinical usefulness of this parameter, allowing for the differentiation of the above-mentioned study groups. ROC curve analysis for the group of patients with severe COVID-19 and the healthy control group (Table 3, Figure 2C) showed an AUC of 0.873 (95% confidence interval: 0.814–0.931, *p* = 0.0000, proposed cut-off point: 0.117 AU, with a sensitivity of 87.7% and a specificity of 79.3%), and for the group of convalescents and the control group the AUC was 0.806 (95% confidence interval: 0.726–0.886, *p* = 0.0000, proposed cut-off point: 0.503 AU, with a sensitivity of 60.4% and a specificity of 90.8%) (Table 3, Figure 2D), indicating that this parameter has moderate clinical utility in differentiating the above study groups.

The relative reactivity of AGP N-glycans with the sialo-specific lectin MAA in the group of patients with severe COVID-19 was on average 0.018 ± 0.019 AU (median: 0.013 AU). In the group of convalescents, the reactivity of AGP glycans with this lectin was on average 0.011 ± 0.009 AU (median: 0.009 AU), and in the control group of healthy subjects, this reactivity was on average 0.022 ± 0.030 AU (median: 0.013 AU). The relative reactivity of AGP glycans with MAA differed significantly only between the group of convalescents and the group of healthy controls (*p* = 0.001795) (Table 1, Figure 3A). Spearman’s rank correlation test revealed no significant correlation between the relative reactivities of AGP glycans with the sialo-specific lectin MAA and the other parameters studied (*p* > 0.05 for each comparison).

The AUC value for this parameter obtained in the ROC curve analysis performed for the convalescent group and the healthy control group was 0.664 (95% confidence interval: 0.563–0.764, *p* = 0.0014, proposed cut-off value: 0.017 AU, with a sensitivity of 83.3% and a specificity of 40.0%) (Table 3, Figure 3B), indicating limited clinical utility of this parameter in differentiating healthy individuals from convalescents.

### 2.3. AGP Fucosylation

The profile and degree of serum AGP fucosylation were analyzed by lectin-ELISA using two fucose-specific lectins: LTA, which detects antennary fucose linked via an α1,3-linkage to GlcNAc (Lewis^x^ structures, Le^x^), and AAL, which detects antennary fucose linked via an α1,2-linkage to Gal and via an α1,3- or α1,4-linkage to GlcNAc, as well as core fucose α1,6-linked.

The relative reactivity of AGP glycans with LTA was 0.135 ± 0.076 AU (median: 0.116 AU) in the group of patients with severe COVID-19, 0.191 ± 0.078 AU (median: 0.173 AU) in the group of convalescents, and 0.255 ± 0.116 AU (median: 0.245 AU) in the control group of healthy subjects. A significant reduction in the relative reactivity of AGP glycans with LTA was demonstrated in the group of patients with severe COVID-19, both compared to the group of convalescents (*p* = 0.000018) and the healthy control group (*p* = 0.000000). This parameter was also significantly decreased in the convalescent group compared to the control group (*p* = 0.000990) (Table 1, Figure 4A).

Spearman’s rank correlation test revealed a high negative correlation between the relative reactivities of AGP glycans with LTA and the AGP concentrations in the tested sera (r = −0.53, *p* = 0.000000), a moderate positive correlation between the relative reactivities of AGP glycans with LTA and the relative reactivities of AGP glycans with sialo-specific SNA as mentioned in Section 2.2, and a weak positive correlation between the relative reactivities of AGP glycans with LTA and the relative reactivities with AAL (r = 0.20, *p* = 0.004332) (Table 2).

For the relative reactivities of AGP glycans with LTA, the AUC value calculated based on ROC curve analysis for the group of patients with severe COVID-19 and convalescents was 0.724 (95% confidence interval: 0.638–0.809, *p* = 0.0000, proposed cut-off point was 0.126 AU, with a sensitivity of 81.3% and a specificity of 58.6%), for the group of patients with severe COVID-19 and the healthy control group, the AUC value was 0.832 (95% confidence interval: 0.765–0.899, *p* = 0.0000, proposed cut-off point was 0.19 AU, with a sensitivity of 75.4% and a specificity of 79.3%), which indicates the average clinical utility of this parameter for differentiating these groups; whereas for the convalescent and control groups, the AUC value was 0.682 AU (95% confidence interval: 0.584–0.781, *p* = 0.0003, proposed cut-off point was 0.222 AU, with sensitivity of 68.8% and specificity of 63.1%) (Table 3, Figure 4B–D), which indicates a limited clinical utility of this parameter for distinguishing these groups.

The relative reactivity of AGP glycans with AAL in the group of patients with severe COVID-19 was 0.114 ± 0.076 AU (median: 0.099 AU). In the convalescent group, the relative reactivity with this lectin was 0.123 ± 0.059 AU (median: 0.116 AU), and in the control group, the mean value was 0.119 ± 0.043 AU (median: 0.114 AU). No significant differences were found in the relative reactivities of AGP glycans with AAL between the analyzed groups (*p* > 0.05) (Table 1, Figure 5), and that is why ROC curve analysis was not performed. The nonparametric Spearman rank correlation test revealed a weak negative correlation between the relative reactivities of serum AGP glycans with AAL and AGP concentrations (see Section 2.1) and a weak positive correlation between the relative reactivities of AGP glycans with AAL and the relative reactivities with SNA as mentioned in Section 2.2, as well as with LTA relative reactivity as mentioned above in this section (Table 2).

### 2.4. Serum Oxidation-Reduction Potential

The measured static oxidation-reduction potential in the group of patients with severe COVID-19 was 160.2 ± 17.2 mV (median: 161.8 mV), in the group of convalescents it was 181.9 ± 13.7 mV (median: 182.3 mV), and in the healthy control group it was 180.0 ± 14.1 mV (median: 179.1 mV). The sORP values in the sera of patients with severe COVID-19 were significantly reduced compared to both the convalescents (*p* = 0.000000) and healthy controls (*p* = 0.000000). No significant differences were found between the convalescents group and the control group (*p* > 0.05) (Table 1, Figure 6A).

Spearman’s rank correlation test revealed a moderate negative correlation between sORP values and AGP concentrations only (see Section 2.1), a weak positive correlation between sORP and AGP relative reactivity with SNA (see Section 2.2), and a weak negative correlation between cORP and AGP relative reactivity with MAA (see Section 2.2) (Table 2).

The ROC curves for sORP are presented in Figure 6B,C. For the group of patients with severe COVID-19 and the group of convalescents, the AUC value calculated after ROC curve analysis was 0.849 (95% confidence interval: 0.772–0.926, *p* = 0.0000, proposed cut-off point was 166.6 mV, with sensitivity of 91.7% and specificity of 68.8%), while for the group of patients with severe COVID-19 and the group of healthy controls, the calculated AUC value was 0.834 (95% confidence interval: 0.753–0.916, *p* = 0.0000, proposed cut-off point was 166.3 mV, with sensitivity of 89.6% and specificity of 66.7%) (Table 4), which indicates the average clinical usefulness of this parameter for differentiating the above-mentioned groups of study participants.

The measured capacitive redox potential in the group of patients with severe COVID-19 was 0.14 ± 0.12 μC (median: 0.11 μC), in the group of convalescents, it was 0.11 ± 0.02 μC (median: 0.10 μC), and in the control group, the cORP level was 0.10 ± 0.01 μC (median: 0.10 μC). The cORP values were significantly elevated in the group of patients with severe COVID-19 compared to the group of healthy controls (*p* = 0.005866). There were no significant differences in cORP values between the severe COVID-19 patients and the convalescents, as well as between convalescents and the healthy subjects (*p* > 0.05) (Table 1, Figure 7A). No significant correlations were observed between cORP and the values of other examined parameters.

ROC curve for cORP values is presented in Figure 7B. For the group of patients with severe COVID-19 and the group of healthy control, the AUC value calculated after ROC curve analysis was 0.663 (95% confidence interval: 0.552–0.775, *p* = 0.0040, proposed cut-off point was 0.11 μC, with sensitivity of 95.8% and specificity of 37.5%), which indicates limited usefulness of this parameter for differentiating between these groups of study participants (Table 4).

## 3. Discussion

A comparison of the obtained AGP concentration values between the three analyzed study groups clearly shows that AGP concentration values significantly increase during the course of inflammation accompanying severe COVID-19, and after its resolution (the group of recovered patients) they decrease to values considered physiological, which is consistent with previous reports on the role of AGP in the development of inflammation associated with the course of many inflammatory diseases, including infectious diseases [11,12,13,14]. Beimdiek et al. obtained serum AGP concentration results very similar to those presented in this study in patients with severe and moderately severe COVID-19 (median AGP concentration in COVID-19: 167 mg/dL, compared to a median of 68.9 mg/dL in the control group) [15]. This is consistent with the current state of knowledge, as AGP is a positive acute-phase protein, the synthesis of which is induced, among others, by inflammatory mediators, such as proinflammatory interleukins 1, 6, and 11 and TNF-α, synthesized during the inflammatory reaction [3,5,6]. Hochepied et al. suggested that the increase in serum AGP concentration is part of a negative feedback loop aimed at modulating the growing immune response [16].

ROC curve analysis demonstrated high clinical utility of AGP concentration measurements in differentiating patients with severe COVID-19 from both recovered and healthy individuals. However, it should be noted that this is a nonspecific marker whose concentration increases regardless of the causes of the developing inflammation. Given that one of the exclusion criteria for inclusion of a study participant in the recovered or healthy control group was the lack of any inflammatory illnesses, including non-infectious ones, it is difficult to assess whether the differences in serum AGP concentrations between these groups and the group of patients with severe COVID-19 would have been so pronounced had this exclusion criterion not been applied. Therefore, measuring serum AGP concentration alone is insufficient to definitively diagnose severe COVID-19. The methodology used also does not allow for assessing the prognostic value of AGP concentrations during severe COVID-19—the study excluded individuals with mild and moderately severe disease, so we do not know what the values of this parameter would be in these patient groups. On the other hand, Beimdiek et al. demonstrated in their study that there are no significant differences in AGP concentrations between patients with moderately severe and severe COVID-19 (in moderately severe COVID-19, the median value was 150 mg/dL, while in severe COVID-19, the median value was 173.5 mg/dL) [15], and the AGP concentration values we obtained for the group of patients with severe COVID-19 (median: 166.81 mg/dL) are similar to the values reported by the authors. In the present study, the proposed cut-off value for differentiation between patients with severe COVID-19 and convalescents was 99.612 mg/dL (sensitivity 85.1%, specificity 97.9%), and it was 95.893 mg/dL when COVID-19 patients were differentiated from healthy subjects (sensitivity 96.9%, specificity 86.9%). Including patients with varying degrees of SARS-CoV-2 infection severity would allow for drawing additional valuable conclusions, which should be considered in future studies. To sum up, it can be said that serum AGP concentrations, although not a specific marker for the severe course of COVID-19, may be an additional parameter reflecting the severity of this viral disease and the recovery process.

The present studies demonstrated a decreased degree of α2,6-sialylation of AGP N-glycans in the group of patients with severe COVID-19 compared to the healthy control group, and an increased degree of α2,6-sialylation in the group of convalescents compared to the control group and COVID-19 patients as well. This parameter, expressed as the relative reactivity of AGP glycans with SNA, very strongly negatively correlates with AGP concentration. On the other hand, the expression of MAA-reactive sialic acid α2,3-linked differed significantly only between the control group and the convalescent group, with significantly decreased expression in the latter group. It should be noted, however, that the reactivities of AGP N-glycans with this lectin were very low in all study groups, which may increase the error rate in the final result of the statistical analysis, but also suggests a small contribution of terminal sialic acid attached via an α2,3-linkage to the oligosaccharide antenna of serum AGP glycans, which is consistent with previous reports by other authors [17].

The two types of sialylations of AGP N-glycans mentioned above, although chemically differing only in the sialic acid-Gal binding site of the oligosaccharide antenna, have far-reaching molecular and functional consequences. Only the attachment of sialic acid to AGP glycans via an α2,3-linkage allows for the formation of the sLe^x^ (sialo-Lewis^x^) epitope, important for regulating the immune response. This structure is also important for other plasma/serum proteins. Complement factor H, which inhibits the complement activation cascade and is crucial for the recognition of self-antigens, binds α2,3-sialylated glycans present on host cells [18]. Interestingly, Qin et al. [19] demonstrated that severe COVID-19 is associated with increased α2,6-sialylation compared to both healthy individuals (*p* < 0.0001) and patients with mild COVID-19 (*p* < 0.01), but this study examined the entire plasma glycoproteome, particularly complement proteins, as well as lung tissue samples. Furthermore, the researchers isolated a fraction of 44 proteins with high SNA reactivity from pooled plasma and subsequently identified these proteins; AGP was not found among them [19]. The results presented in this paper for AGP are inconsistent with those reported by Qin et al. [19], as we have observed that AGP reactivity with SNA significantly decreases during severe COVID-19 when compared with other study groups. However, differences in the assumed research goals and methodological approaches, also related to the sensitivity and specificity of the research methods used, may be the cause of the observed discrepancies in the obtained results.

ROC curve analysis demonstrated high clinical utility of the relative reactivity of AGP glycans with SNA in differentiating between patients with severe COVID-19 and convalescents (proposed cut-off point was 0.187 AU with sensitivity 100% and specificity 86.2%), and moderate clinical utility in differentiating between patients with severe COVID-19 and healthy individuals (proposed cut-off point was 0.117 AU with sensitivity 87.7% and specificity 79.3%), as well as between convalescents and healthy individuals (proposed cut-off point was 0.503 AU with sensitivity 60.4% and specificity 90.8%). On the other hand, the relative reactivity of serum AGP glycans with MAA is characterized by limited clinical utility, being significant only in distinguishing between groups of convalescents and healthy individuals. However, the previously mentioned low reactivities of serum AGP glycans with this lectin should be kept in mind. Considering the above, it should be emphasized that the results of the analyses presented by us indicate AGP as a glycoprotein closely associated with severe COVID-19, even though it is not a marker specific to this disease.

The observed lack of significant differences in AGP glycans reactivity with fucose-specific AAL between studied groups, in contrast to AGP relative reactivity with LTA (a weak positive correlation was observed between relative reactivities of AGP glycans with these two lectins), may be due to the lectin’s overly broad specificity, masking significant differences in its reactivity, or to the limited access of AGP glycans to AAL in severe COVID-19, which may be due to conformational differences in this protein, associated with the course of this viral disease and the limited access of glycans that specifically react with AAL. However, this is only a hypothesis that should be checked in future studies.

Despite some overlap in the specificity of AAL and LTA, the relative reactivity of AGP glycans with LTA differed significantly between the study groups, with the lowest values in the group of patients with severe COVID-19, the highest in the group of healthy individuals, and intermediate in the group of convalescents. The results obtained seem to be consistent with those presented by Paton et al. [20], who demonstrated a reduced degree of fucosylation of blood plasma N-glycans in patients with severe COVID-19, observing also that the degree of fucosylation negatively correlates with the severity of the disease. Based on their study results, the authors proposed a potential glycobiomarker such as fucosylated, biantennary complex N-glycan with the composition Fuc1Hex5HexNAc5 (1 fucose molecule, 5 hexose molecules, 5 N-acetylhexose molecules), which could be used for prognostic purposes [20]. However, it should be noted that the studies mentioned above examined all plasma glycome, without identification of individual glycoproteins. Furthermore, they were conducted using another research method (LC-MS/MS), the results of which provide different information on the glycosylation of glycoproteins, focusing on the quantitative and qualitative composition of the analyzed glycans. On the other hand, lectin-ELISA allows, to some extent, reflection of the bioavailability of individual sugar/oligosaccharide residues to endogenous ligands. In summary, it can be said that studies analyzing serum AGP glycosylation in severe COVID-19 are innovative and warrant further investigation on a broader scale.

The results of ROC curve analysis show that the expression of Le^x^ structures in AGP glycans, has a potential as glycomarker with moderate clinical utility for differentiation severe COVID-19 patients from convalescents (proposed cut-off point was 0.129 AU with sensitivity 81.3% and specificity 58.6%) and healthy subjects (proposed cut-off point was 0.19 AU with sensitivity 75.4% and specificity 79.3%), as well as between convalescents and heathy individuals (proposed cut-off point was 0.222 AU with sensitivity 68.8% and specificity 63.1%). It should also be mentioned that Le^x^ expression in AGP glycans is highly negatively correlated with AGP concentration and moderately positively associated with expression of SNA-reactive sialic acid. The above observations reveal a characteristic pattern of serum AGP glycosylation in severe COVID-19, reflecting abnormalities related to the biological role of this glycoprotein during inflammation, which is also an inherent attribute of SARS-CoV-2 infection. The significantly elevated AGP concentration, typical for the acute-phase reaction, associated with decreased expression of SNA-reactive sialic acid and Lex oligosaccharide structures (these parameters correlated moderately positively), indicates that the molecular mechanisms responsible for the proper glycosylation of AGP may be disturbed as the disease progresses. To determine whether, or to what extent, this hypothesis is true, further studies should be conducted focused on detailed AGP N-glycans composition, as well as the mechanisms of reactions that occur between AGP glycans and their endogenous ligands during COVID-19 development and progression.

The measured sORP values in the group of patients with severe COVID-19 were significantly reduced compared to both the convalescents and the healthy control group. The opposite was observed for cORP values, which were significantly elevated in the group of patients with severe COVID-19 in comparison to the healthy subjects and visibly increased vs. convalescents, insignificantly, however. The observed differences are logical, given the nature of these parameters, as higher cORP values reflect a greater proportion of available reducing agents in the tested sample, and thus a greater ability to mitigate oxidative stress and lower oxidant activity, expressed by sORP. On the other hand, the reduction in sORP values in the group of patients with severe COVID-19 is surprising, considering the role of oxidative stress in the pathophysiology of this disease. However, the observed significant negative correlation between this parameter and AGP concentrations suggests the antioxidant effect of AGP, enhanced at high concentrations of this glycoprotein, which may be one of the agents responsible for such a result. To verify the above hypothesis, further studies should be done in which the levels of other proteins that have antioxidant effects will be correlated with sORP values measured with similar study groups. Interestingly, the results obtained in the present study, which were focused on measuring redox potential, are consistent with those obtained by Krasic et al. [21] in a study of 67 children with multisystem inflammatory syndrome caused by earlier SARS-CoV-2 infection, who were treated with methylprednisolone. The authors demonstrated elevated ORP levels (median: 188.0 mV) during multisystem inflammatory syndrome, which subsequently decreased after methylprednisolone administration (median: 176.3 mV, *p* = 0.04), and this condition persisted for 6 months [21]. Our observations are like this reported by Krasic et al. [21], as in severe COVID-19 the sORP values were significantly decreased (median: 161.8 mV) when compared to convalescents (182.3 mV) and healthy controls (179.1 mV). However, we do not have complete information on the previous medications taken by patients with severe COVID-19, so it is not possible to compare the influence of the treatment regimen for SARS-CoV-2 infection on sORP values in this group of participants. Even though the authors conducted their study on a group of participants who differ in age from severe COVID-19 patients examined by us, Krasic et al. [21] and our observations regarding the tendency to reduce sORP values after treatment of patients with severe SARS-CoV-2 infection are convergent. The authors also mentioned that methylprednisolone treatment led to a rapid increase in antioxidant defense enzymes in erythrocytes (super oxide dismutase, SOD and catalase, CAT) resulting in clinical and echocardiographic improvement [21] which may additionally explain why sORP in severe COVID-19 patients (certainly intensively treated) significantly decreased in comparison to convalescent and healthy subjects. We cannot also exclude that patients with severe COVID-19 took increased doses of antioxidant supplements, which additionally may negatively influence sORP inter alia via reactive oxygen species neutralization.

The ROC curve analysis shows that this parameter has moderate clinical value to differentiate COVID-19 patients from convalescents (AUC = 0.849) and healthy subjects (AUC = 0.834), with proposed cut-off points 166.6 mV (sensitivity 91.7%, specificity 68.8%) and 166.3 mV, respectively (sensitivity 89.6%, specificity 66.7%). Taking the above into account, it can be said that the sORP level seems to be independent of the treatment method and age of patients with severe SARS-CoV-2 infection, being a useful biomarker when assessing response to therapy and monitoring the recovery process. Methodological differences in sORP measurement by Krasic et al. [21] study and our also do not seem to affect the final conclusions drawn. To the best of our knowledge, these are the first studies presenting levels of redox potential measured in sera of adult patients with severe COVID-19 and in convalescents.

After analyzing the obtained research results and comparing them with previous studies conducted by our team on the analysis of the profile and degree of glycosylation of other serum glycoproteins (immunoglobulin G, IgG, and clusterin, CLU) playing a role in immune response and development of inflammation and/or oxidative stress in the same groups of participants [22,23], interesting observations emerge. In contrast to the glycosylation pattern of serum AGP presented in this article, IgG glycans not only did not exhibit variation in the expression of terminal α2,6-linked sialic acid, but there were also no significant changes in the concentration of this glycoprotein in sera of severe COVID-19 patients compared to convalescents and healthy individuals. However, in the case of fucose expression of Le^x^ structures, a significant increase was observed in patients with severe COVID-19 compared to the other study groups [22]. Interestingly, in the case of CLU, the opposite pattern was observed compared to the differences in serum AGP concentrations and the degree of its sialylation and fucosylation. In patients with severe COVID-19, CLU concentrations were significantly lower, accompanied by a significant increase in the expression of SNA-reactive sialic acid and LTA-reactive fucose on its glycans, compared to the other study participants [23]. These observations further confirm that, despite certain similarities in the biological role of glycoproteins involved in the body’s immune response and the development of inflammation following viral infection, both their concentrations and glycosylation patterns differ throughout the course of this disease. This not only confirms the complexity of the entire process associated with the development of viral infection, but also highlights how much we still have to learn about the interrelationships between various proteins/glycoproteins participating directly or indirectly in the spread of viral infection, as well as the molecular mechanisms regulating these interrelationships, including those related to the interactions between the glycans of glycoproteins and their endogenous ligands.

## 4. Materials and Methods

### 4.1. Patient Samples

The study was conducted following the Helsinki-II declaration, and the protocol was approved by the Bioethics Committee of the Medical University of Bialystok (Permission No. APK.002.26.2021, APK-002.171.2023, annexed on 20 February 2025). Informed written consent was obtained from each study participant. The project included patients with severe COVID-19 who were conscious and able to make decisions about participation in the study. The serum samples were collected from patients who were admitted to the Emergency Department of the University Clinical Hospital in Bialystok between January and November 2021 with active SARS-CoV-2 infection, with diagnosis confirmed using a polymerase chain reaction (PCR) test (n = 87; age 56–75). The group of COVID-19 patients included subjects at 3rd and 4th stages based on Modified Early Warning Score (MEWS) classification (Table 5) who required intensive treatment because of pneumonia, with or without acute respiratory distress syndrome (ARDS), and with or without multiple organ dysfunction syndrome (MODS) [24]. None of the SARS-CoV-2-infected patients were in the 1st or 2nd stage of the disease (exclusion criteria). The Polish Society of Epidemiology and Infectious Diseases recommended the MEWS for identifying the stage of COVID-19, which relies on the following parameters: body temperature, blood pressure, heart and respiratory rates, and neurological symptoms [24]. Based on the above parameters, four stages of COVID-19 progression were described: (1) asymptomatic and mild symptomatic infection, (2) symptomatic infection with pneumonia, without symptoms of ARDS, (3) symptomatic infection with pneumonia and symptoms of ARDS, and (4) symptomatic infection with MODS (Table 1). The characteristics of patients with severe COVID-19 are presented in Table 6.

The convalescents’ group was composed of 48 subjects (age 28–75, 18 males/30 females), with positive anti-SARS-CoV-2 IgG antibodies in blood, who had suffered from SARS-CoV-2 infection in the last 3–4 weeks before recruitment to the study, did not take any medications, and for whom the course of the disease was mild (increased temperature, loss of taste and smell, headache, fatigue, and muscle pain). None of the patients required hospitalization, and they were SARS-CoV-2-negative at the time of collecting blood serum for the present study. Convalescents who qualified for the study did not receive any anti-inflammatory drugs at the time of blood collection for the study. The control group consisted of 65 healthy participants (age 30–74, 27 males/38 females) who did not suffer from SARS-CoV-2, and whose blood was free from specific IgG antibodies against this virus. Both convalescent and healthy subjects from the control group with comorbidities were excluded from participation in the study.

### 4.2. Biological Samples

From all study participants, 5.5 mL of venous blood was collected in test tubes without anticoagulants (S-Monovette, SARSTEDT, Nümbrecht, Germany). Next, within 30 min, blood was centrifuged for 20 min at 1000× *g* to obtain serum, which was further divided into smaller portions and stored at −75 °C at the Department of Clinical Laboratory Diagnostics, Medical University of Bialystok. Blood serum samples transported from the Medical University of Bialystok were stored at −86 °C at the Wroclaw Medical University Biobank until the start of the research. Dividing the samples into smaller portions allowed us to avoid repeated thawing and freezing of the same sample, which could influence the values of the analyzed oxidative stress parameters [25]. Before performing the assay, all serum samples were gradually defrosted and mixed.

### 4.3. AGP Concentration

AGP concentrations in the tested samples were determined via an immunoturbidimetric method in the Konelab20i^®^ biochemical analyzer (ThermoScientific, Vantaa, Finland), and compatible reagents from Sentinel Diagnostics (Milan, Italy). A five-level Immuno-Multical calibrator set from Cormay Diagnostics (Łomianki, Poland) was used to generate the standard curve with AGP concentration in the range 13–200 mg/dL, along with 0.9% sodium chloride solution as a blank.

### 4.4. Lectin-ELISA

The ELISA plate wells (Nunc MaxiSorp, Thermo Fisher Scientific, Glostrup, Denmark) were coated with goat anti-human AGP polyclonal antibodies (Invitrogen, Thermo Fisher Scientific, catalog no. PA1-26903; Rockford, IL, USA) diluted 1:1500 in 10 mM TBS, pH = 8.5. After 2 h incubation at 37 °C, the plate was washed three times using the same buffer. Due to the high absorbance of blanks in the case of AAL reactivity observed in the preliminary experiments, oxidation of oligosaccharides of anti-human AGP polyclonal antibodies, which coated the ELISA plate, was performed. Sodium meta-periodate solution (100 mM NaIO_4_, 100 mM NaHCO_3_, pH = 8.1) was added, and after a 90-min incubation in the dark at room temperature, the plate was washed five times with 10 mM TBS, pH = 7.5. For the remaining lectins, this step was omitted. In the next step, free binding sites of the ELISA plate wells were blocked by 10 mM TBS, 0.1% Tween20, 1% BSA (blocking buffer, pH = 7.5). After 2 h of incubation at 37 °C, plates were stored at 4 °C overnight. The serum samples were diluted in 10 mM TBS 0.1% Tween20 to obtain 500 ng AGP/100 µL, applied to each well of the ELISA plate, and incubated at 37 °C for two hours with gentle shaking. All samples were analyzed in duplicate to minimize imprecision. For each lectin-ELISA experiment, two pairs of blanks and internal control sera with known lectin reactivity were included. Blanks contained all reagents, but instead of patient samples, 10 mM TBS, 0.1% Tween20, pH = 7.5 (washing buffer) was used. After each step of lectin-ELISA, the wells were washed using a washing buffer. In the next step, biotinylated lectins (Vector Laboratories Inc., Burlingame, CA, USA) detecting terminal α2,6- and α2,3-linked sialic acid (SNA and MAA, respectively), diluted in a ratio of 1:1000 for SNA, 1:250 for MAA and LTA, and 1:750 for AAL, were used (the exact specificities of lectins are presented in Table 7). Then, the plates were incubated for one hour at 37 °C with gentle shaking. To detect the AGP–lectin complexes, ExtrAvidin alkaline phosphatase labeled (Sigma-Aldrich, catalog no. E2636; Saint Louis, MO, USA), diluted in a ratio of 1:10,000 in the washing buffer, was used. Next, plates were incubated for one hour at 37 °C, and then, the color reaction with disodium para-nitrophenyl phosphate was induced. The absorbances were measured with Mindray MR-96A Microplate Reader (Mindray Bio-Medical Electronics, Shenzen, China) at 405 nm with a reference filter of 630 nm. The relative reactivities of AGP glycans with specific lectins were expressed in absorbance units (AU), after subtracting the absorbances of the blank samples. The lectin-ELISA method used in the present study for AGP glycosylation analysis was based on the method described by us previously for AGP and its modification for clusterin as well [26,27,28,29].

### 4.5. ORP Measurement

The redox potential of the sera in all three study groups was measured on a MiOXSYS C+^®^ device using disposable MiOXSYS sensor^®^ measuring cassettes (Caerus Biotechnologies, Vilnius, Lithuania). The method is based on measuring the redox potential of a platinum electrode, where oxidation and reduction reactions occur after the introduced sample completes an electrical circuit. Assessment of the balance between the formation of reactive oxygen species and their removal from the body, based on the electrical potential associated with the flow of electrons between chemical substances. The measured potential, expressed in mV, reflects the total content of all oxidants and antioxidants contained in the sample, thus reflecting the degree of oxidative stress [34].

Forty-eight samples from each group of study participants were selected for the assays, with samples selected so that the highest, lowest, and medium AGP concentrations were represented in equal proportions (16 + 16 + 16) in each group. Before starting the redox potential determinations in the examined samples, the apparatus was calibrated using a calibration verification key. The mV of sORP readings should fall within the ranges of 99.0–101.0 mV (lower measurement range) and 295.8–304.2 mV (upper measurement range). After calibration, measurements of the test samples began. A disposable measurement cassette was inserted into the analyzer, and 30 µL of the sample was pipetted immediately onto the test field. The signal was read 5 min after the sample application.

### 4.6. Statistical Analysis

The obtained results were statistically analyzed using STATISTICA 13.3PL (StatSoft, Krakow, Poland). The normality of the distribution of the obtained values of the examined parameters in each study group was analyzed using the Shapiro-Wilk test, which demonstrated normal distribution for AGP concentration values in the group of patients with severe COVID-19 and convalescents, for the reactivity of AGP glycans with SNA in the convalescent group, and for the sORP in each study group. The values of the remaining parameters were not normally distributed.

To determine whether there were significant differences between the study groups in AGP concentration values, the reactivity of its glycans with individual lectins, and the values of the capacitive redox potential, the nonparametric Mann-Whitney U test was used. ANOVA, supplemented with Tukey’s post hoc test, was used to compare static redox potential values between groups. Differences with a significance coefficient less than 0.05 (*p* < 0.05) were considered significant.

Because not all parameter values were normally distributed, the nonparametric Spearman’s rank test was used to determine whether correlations existed between them. The strength of correlation was interpreted based on Spearman’s rank coefficient (r): 0.1 ≤ r < 0.3—weak correlation, 0.3 ≤ r < 0.5—moderate correlation, 0.5 ≤ r < 0.7—high correlation, 0.7 ≤ r < 0.9—very high correlation, 0.9 ≤ r < 1.0—almost perfect correlation.

Next, for parameters whose values differed significantly between the study groups, a ROC analysis was performed, which allowed for the determination of the AUC, the calculation of sensitivity and specificity for each of the analyzed parameters, and the proposal of an optimal cut-off point. The Youden index, a measure of diagnostic test quality, was used to determine the cut-off points for the analyzed parameters. The equilibrium point lies at the intersection of the ROC curve with the line TPR (True-Positive Rate, sensitivity) = 1 − FPR (False-Positive Rate, specificity) = TNR (True-Negative Rate) and represents the “cut-off” point for which the classifier achieves a sensitivity = specificity balance. The optimal cut-off point is the threshold value of the probability or predictor that provides a compromise between the model’s sensitivity and specificity. The AUC value was used to assess the clinical usefulness of the tested parameters in differentiating between study participant groups: 0.0 ≤ AUC < 0.5—zero test value, 0.5 ≤ AUC < 0.7—limited test value, 0.7 ≤ AUC < 0.9—moderate test value, and 0.9 ≤ AUC—high test value.

## 5. Conclusions

To the best of our knowledge, presented in this article, the results of serum AGP glycosylation analyses in severe SARS-CoV-2 infection and convalescents, combined with redox potential measurement, are the first such reports. The results allowed us to draw several conclusions. It was not a surprising observation that significantly higher serum AGP concentrations in patients with severe COVID-19 compared to convalescents and healthy individuals confirm the involvement of the AGP molecule in the acute-phase reaction accompanying this viral disease. This parameter is characterized by high diagnostic value, with very high sensitivity and specificity, for differentiating patients with severe COVID-19 from convalescents and healthy individuals. Moreover, AGP levels significantly negatively correlated with the degree of its α2,6-sialylation, antennary α1,3-fucosylation, and the level of sORP, which is a characteristic image of the expression of these parameters for the course of severe COVID-19.

What is interesting is that the expression of α2,6-linked sialic acid in AGP glycans was significantly highest in the convalescents group when compared not only with severe COVID-19 patients but also with healthy subjects. This parameter has high diagnostic utility for distinguishing between severe COVID-19 patients and convalescents, and moderate diagnostic utility for distinguishing between healthy individuals from convalescents and severe COVID-19 patients. The expression of α2,6-linked sialic acid also significantly positively correlated with the relative reactivities of AGP glycans with fucose-specific LTA. These observations support the previous conclusion that severe COVID-19 is manifested by elevated AGP concentrations accompanied by reduced α2,6-sialylation of its glycans, while the reduction in AGP levels observed during the recovery process is associated with a significant increase in the degree of α2,6-sialylation.

The relative reactivity of AGP glycans with LTA was significantly reduced in patients with severe COVID-19 vs. convalescents and healthy subjects, which may indicate an association between the severe course of SARS-CoV-2 infection and the expression of Le^x^-type oligosaccharide structures on AGP glycans. This is puzzling because during the acute phase, which undoubtedly accompanies severe COVID-19, typical for serum AGP glycans, Le^x^-type antennary fucosylation should increase. In connection with the above, we may be dealing here with a change in the antennary fucosylation profile of serum AGP glycans during SARS-CoV-2 infection (Le^a^? Le^b^?), rather than a decrease in fucose expression. This hypothesis should be tested in future studies. What is interesting is that the relative reactivity of AGP glycans with fucose-specific AAL, a lectin with a much broader specificity than LTA, did not differ significantly between the study groups.

Serum sORP values were significantly reduced in patients with severe COVID-19 compared to convalescents and healthy individuals. The observed negative correlations between sORP values and AGP levels may suggest an antioxidant effect of AGP in diseases with increased oxidative stress, such as severe COVID-19. Because both AGP and sORP levels are parameters whose measurement is relatively fast, these two parameters have a chance to become additional diagnostic biomarkers of severe SARS-CoV-2 infection, also useful in monitoring the recovery process after treatment, which makes them useful in clinical practice.

Concluding, the research results we obtained allowed us to propose a set of four biomarkers with potential diagnostic and/or clinical applications for monitoring severe COVID-19 progression, such as AGP concentration, expression of terminal sialic acid α2,6-linked, expression of fucose of Le^x^ sugar structures in AGP glycans, as well as sORP measurement. The biological role of serum AGP in severe COVID-19 remains unclear, particularly in the context of the role of its N-glycans in the spread/persistence of SARS-CoV-2 viral infection. A similar situation occurs in the case of serum redox potential measurement and considering this parameter as a marker of oxidative stress usable in the diagnostics of viral diseases. The results of the analyses presented in this article indicate the need for further, in-depth research on the importance of examined parameters in the immune response, their diagnostic and clinical potential, and the possibility of their use in monitoring the course of viral infection and the recovery process. The molecular interactions via glycans between serum glycoproteins and their endogenous ligands should also be considered in such examinations.

## Figures and Tables

**Table 1:** Obtained values of examined serum parameters in individual patient groups.

	**Group**	**COVID-19** **n = 87**	**Convalescents** **n = 48**	**Healthy Control** **n = 65**
**Parameter**				
* AGP [mg/dL]		165.06 ± 58.96 (166.81)	61.65 ± 21.77 (60.53) p^1^ = 0.000000	63.50 ± 21.39 (63.08) p^1^ = 0.000000
* SNA [AU]		0.110 ± 0.160 (0.066)	0.553 ± 0.259 (0.529) p^1^ = 0.000000	0.289 ± 0.168 (0.268) p^1^ = 0.000000 p^2^ = 0.000000
* MAA [AU]		0.018 ± 0.019 (0.013)	0.011 ± 0.009 (0.009)	0.022 ± 0.030 (0.013) p^2^ = 0.001795
* LTA [AU]		0.135 ± 0.076 (0.116)	0.191 ± 0.078 (0.173) p^1^ = 0.000018	0.255 ± 0.116 (0.245) p^1^ = 0.000000 p^2^ = 0.000990
AAL [AU]		0.114 ± 0.076 (0.099)	0.123 ± 0.059 (0.116)	0.119 ± 0.043 (0.114)
	**Group**	**COVID-19** **n = 48**	**Convalescents** **n = 48**	**Healthy Control** **n = 48**
**Parameter**				
** sORP [mV]		160.2 ± 17.2 (161.8)	181.9 ± 13.7 (182.3) p^1^ = 0.000000	180.0 ± 14.1 (179.1) p^1^ = 0.000000
* cORP [µC]		0.14 ± 0.12 (0.11)	0.11 ± 0.02 (0.10)	0.1 ± 0.01 (0.10) p^1^ = 0.005866

The table presents the means and standard deviations (SD) for the analyzed parameter values in each study group. Median values are given in parentheses. n—number of study participants in each group, AU—absorbance unit, sORP—static redox potential; cORP—capacitive redox potential; *p*—significance coefficient (probability value). *—significant differences (*p* < 0.05) in the Mann-Whitney U test; **—significant differences (*p* < 0.05) in Tukey’s U test, compared to: p^1^—patients with severe COVID-19, p^2^—convalescents.

**Table 2:** Correlations between the tested parameters.

Parameters	*r*	*p*	Correlation
**AGP/SNA**	**−0.76**	**0.000000**	**Very high, negative**
**AGP/LTA**	**−0.53**	**0.000000**	**High, negative**
AGP/AAL	−0.28	0.000050	Weak, negative
AGP/sORP	−0.36	0.000009	Moderate, negative
SNA/LTA	0.41	0.000000	Moderate, positive
SNA/AAL	0.18	0.008836	Weak, positive
LTA/AAL	0.20	0.004332	Weak, positive
SNA/sORP	0.26	0.001391	Weak, positive
MAA/cORP	−0.26	0.001731	Weak, negative

The table presents significant correlations (*p* < 0.05) between the studied parameters, with a summary of their strength and direction. *p*—significance coefficient (probability value); *r*—Spearman’s rank coefficient; sORP—static redox potential; cORP—capacitive redox potential; SNA—*Sambucus nigra* agglutinin; MAA—*Maackia amurensis* agglutinin; LTA—*Lotus tetragonolobus* agglutinin; AAL—*Aleuria aurantia* lectin. Parameters for which the calculated correlations were high and very high are bolded. The specificity of lectins is given in Section 4.

**Table 3:** Results of ROC curve analysis for AGP concentration values and their reactivity with lectins.

Parameter	AUC (95% CI)	Cut-Off Point	Sensitivity	Specificity	*p*
AGP ^1^ concentration [mg/dL]	**0.949** (0.913–0.986)	99.612	85.1%	97.9%	0.0000
AGP ^2^ concentration [mg/dL]	**0.945** (0.905–0.985)	95.893	96.9%	86.9%	0.0000
SNA ^1^ [AU]	**0.960** (0.926–0.993)	0.187	100%	86.2%	0.0000
SNA ^2^ [AU]	**0.873** (0.814–0.931)	0.117	87.7%	79.3%	0.0000
SNA ^3^ [AU]	**0.806** (0.726–0.886)	0.503	60.4%	90.8%	0.0000
MAA ^3^ [AU]	0.664 (0.563–0.764)	0.017	83.3%	40.0%	0.0014
LTA ^1^ [AU]	**0.724** (0.638–0.809)	0.129	81.3%	58.6%	0.0000
LTA ^2^ [AU]	**0.832** (0.765–0.899)	0.19	75.4%	79.3%	0.0000
LTA ^3^ [AU]	0.682 (0.584–0.781)	0.222	68.8%	63.1%	0.0003

The table summarizes the results of the ROC curve analysis for parameters whose values differ significantly between the analyzed groups. AUC—area under the curve; 95% CI—95% confidence interval; SNA—*Sambucus nigra* agglutinin; MAA—*Maackia amurensis* agglutinin; LTA—*Lotus tetragonolobus* agglutinin; *p*—significance coefficient (probability value). ^1^—comparison of the group of patients with severe COVID-19 and convalescents; ^2^—comparison of the group of patients with severe COVID-19 and healthy controls; ^3^—comparison of the group of convalescents and healthy subjects. AUC values > 0.700 are highlighted in bold. Values with *p* < 0.05 were considered significant. The specificity of lectins is given in Section 4.

**Table 4:** Results of ROC curve analysis for the redox potential values.

Parameter	AUC (95% CI)	Cut-Off Point	Sensitivity	Specificity	*p*
sORP ^1^ [mV]	**0.849** (0.772–0.926)	166.6	91.7%	68.8%	0.0000
sORP ^2^ [mV]	**0.834** (0.753–0.916)	166.3	89.6%	66.7%	0.0000
cORP ^2^ [μC]	0.663 (0.552–0.775)	0.11	95.8%	37.5%	0.0040

The table summarizes the results of ROC curve analysis for ORP values that significantly differed between the analyzed groups. AUC—area under the curve; 95% CI—95% confidence interval; sORP—static redox potential; cORP—capacitive redox potential; *p*—significance coefficient (probability value). ^1^—comparison of the group of patients with severe COVID-19 and convalescents; ^2^—comparison of the group of patients with severe COVID-19 and healthy controls. AUC values > 0.700 are highlighted in bold. Values with *p* < 0.05 were considered significant.

**Table 5:** MEWS scale parameters.

Parameter	Points			
	0	1	2	3
Respiratory rate/min	9–14	15–20	21–29 or ≤8	>29
Heart rate/min	51–100	101–110 or 41–50	111–129 or ≤40	>129
Systolic blood pressure, mmHg	101–199	81–100	≥200 or 71–80	≤70
Diuresis, mL/kg b.w./h	−	−	<0.5	0
Body temperature, °C	36.1–38	38.1–38.5 or 35.1–36	≥38.6 or ≤35	−
Neurological symptoms	Conscious patient	Responds to voice	Reacts to pain	No response to stimuli

Modification based on Flisiak et al. [24].

**Table 6:** Characteristics of the group of patients with severe COVID-19.

Patients with Severe COVID-19, n = 87		
Parameter		n (%)
Age (years)	≤55	21 (24%)
	56–75	28 (32%)
	≥76	38 (44%)
Gender	Male	35 (40%)
	Female	52 (60%)
Hospitalization time (days)	≤10	41 (47%)
	11–20	32 (37%)
	≥21	14 (16%)
Comorbidities	Absent	36 (41%)
	Present	51 (59%)
	Hypertension	42 (48%)
	Coronary artery disease	33 (38%)
	Diabetes	28 (32%)
	Obesity	11 (13%)
	Other (cancer)	4 (5%)
**Symptoms**		**n (%)**
Cough	None	9 (10%)
	Present	78 (90%)
Fever	None	13 (15%)
	Present	74 (85%)
Dyspnea	None	14 (16%)
	Present	73 (84%)
Respiratory failure	None	7 (8%)
	Present	80 (92%)

Prepared based on Sołkiewicz et al. [22].

**Table 7:** Lectin specificity.

Lectin	Specificity
SNA (*Sambucus nigra* agglutinin)	detects terminal sialic acid linked to Gal via an α2,6 bond [30]
MAA (*Maackia amurensis* agglutinin)	detects terminal sialic acid attached via an α2,3 bond to the Gal of the sugar antenna [31]
LTA (*Lotus tetragonolobus* agglutinin)	detects antennary fucose attached via an α1,3 linkage to GlcNAc, including sLe^x^ epitopes [32]
AAL (*Aleuria aurantia* lectin)	detects antennary fucose attached via an α1,2-linkage to Gal, α1,3-linkage to GlcNAc, α1,4-linkage to GlcNAc or core fucose attached by α1,6-linkage [33]

Gal—galactose, GlcNAc—N-acetylglucosamine, and sLe^x^—sialo-Lewis^x^.

## Data Availability

All data needed to evaluate the conclusions in the article are present in the article. Additional data related to this study are available on reasonable request from the corresponding author.

## Abbreviations

The following abbreviations are used in this manuscript:

AAL	*Aleuria aurantia* lectin
AGP	α1-acid glycoprotein
AU	Absorbance unit
AUC	Area under the curve
CI	Confidence interval
cORP	capacitive oxidation-reduction potential
COVID-19	coronavirus disease 2019
Fuc	fucose
Gal	galactose
GlcNAc	N-acetylglucosamine
Hex	hexose
IL	interleukin
LC-MS/MS	liquid chromatography with tandem mass spectrometry
LTA	*Lotus tetragonolobus* agglutinin
MAA	*Maackia amurensis* agglutinin
ORM	orosomucoid
ORP	oxidation-reduction potential
p-NPP	para-4-nitro-phenyl-phosphate disodium
ROC curve	receiver operating characteristic curve
ROS	reactive oxygen species
SA	sialic acid
SARS-CoV-2	severe acute respiratory syndrome coronavirus-2
sLe^x^	sialo-Lewis^x^
SNA	*Sambucus nigra* agglutinin
sORP	static oxidation-reduction potential
TNF-α	tumor necrosis factor α
